# Electronic Consultation Services Worldwide: Environmental Scan

**DOI:** 10.2196/11112

**Published:** 2018-12-21

**Authors:** Justin Joschko, Erin Keely, Rachel Grant, Isabella Moroz, Matthew Graveline, Neil Drimer, Clare Liddy

**Affiliations:** 1 CT Lamont Primary Health Care Research Centre Department of Family Medicine University of Ottawa Ottawa, ON Canada; 2 Department of Medicine University of Ottawa Ottawa, ON Canada; 3 Division of Endocrinology/Metabolism The Ottawa Hospital Ottawa, ON Canada; 4 Faculty of Education University of Ottawa Ottawa, ON Canada; 5 Canadian Foundation for Healthcare Improvement Ottawa, ON Canada; 6 Bruyère Research Institute Ottawa, ON Canada

**Keywords:** electronic consultation, interviews, primary care, referral-consultation process, telemedicine, quality of care, specialist care

## Abstract

**Background:**

Excessive wait times for specialist care pose a serious concern for many patients, leading to duplication of tests, patient anxiety, and poorer health outcomes. In response to this issue, many health care systems have begun implementing technological innovations designed to improve the referral-consultation process. Among these services is electronic consultation (eConsult), which connects primary care providers and specialists through a secure platform to facilitate discussion of patients’ care.

**Objective:**

This study aims to examine different eConsult services available worldwide and compare the strategies, barriers, and successes of their implementation in different health care contexts.

**Methods:**

We conducted an environmental scan comprising 3 stages as follows: literature review; gray literature search; and targeted, semistructured key informant interviews. We searched MEDLINE and EMBASE (literature review) and Google (gray literature search). Upon completing the search, we generated a list of potential interview candidates from among the stakeholders identified. Potential participants included researchers, physicians, and decision makers. The maximum variation sampling was used to ensure sufficient breadth of participant experience. In addition, we conducted semistructured interviews by telephone using an interview guide based on the RE-AIM framework. Analyses of transcripts were conducted using a thematic synthesis approach.

**Results:**

A total of 53 services emerged from the published and gray literature. Respondents from 10 services participated in telephonic interviews. The following 4 major themes emerged from the analysis: service structure; benefits of eConsult; implementation challenges; and implementation enablers.

**Conclusions:**

eConsult services have emerged in a variety of countries and health system contexts worldwide. Despite differences in structure, platform, and delivery of their services, respondents described similar barriers and enablers to the implementation and growth and reported improved access and high levels of satisfaction.

## Introduction

Excessive wait times for specialist care pose a serious concern for many patients, leading to duplication of tests, patient anxiety, and poorer health outcomes [1-3]. In response to this issue, many health care systems have begun implementing technological innovations designed to improve the referral-consultation process [4-8]; among these are electronic consultation (eConsult) services—secure Web-based applications that facilitate asynchronous communication between primary care providers (PCP) and specialists, allowing PCPs to ask questions to specialists directly about a patient’s care and, in some cases, avoid the need for a face-to-face consultation.

In 2009, our team launched the Champlain Building Access to Specialists through eConsultation (BASE) eConsult service in the Champlain health region of Ontario. As our service grew, we wanted to gain a better understanding of whether other such services were operating in Canada. To this end, we conducted an environmental scan of services across Canada to ascertain the status of eConsult in each province. Our study found no other eConsult services in the country; only 2 other services emerged besides our own, both of which were exclusively electronic referral (eReferral) systems [9]. Unlike eConsult, which can supplement or replace the in-person referral in some cases, eReferral is simply a platform that lets PCPs submit or schedule patient referrals electronically.

Since then, interest in eConsult has expanded in many countries [7,8]. Champlain BASE has likewise grown, reaching its 50,000th case. Building on its regional success, the service is in the process of expanding province-wide, with money for its implementation earmarked in Ontario’s 2017 budget. In addition, the service is expanding beyond provincial borders. Partnerships with provincial and national groups have resulted in services informed by the BASE model emerging in Alberta, Manitoba, Quebec, and Newfoundland and Labrador.

Given our service’s forthcoming growth, we have endeavored to update our previous scan, making 2 key changes to its scope. First, we have expanded our search for services available outside of Canada to capture a broader range of experiences. Second, we focused our current scan exclusively on eConsult services, as eReferral services address different issues and are not directly comparable to eConsult. These changes allowed us to examine the success and barriers faced by eConsult services in a wide array of different contexts, providing invaluable insight into which elements are most vital and which may—or indeed, should—be adapted to fit the individual circumstances of the region in which they are implemented.

## Methods

### Design

This study follows the methodology used in our previous environmental scan modified to expand from a Canadian to an international focus [9]. Our process was implemented in 3 stages—a literature review, gray literature search, and key informant interviews.

### Population

Our environmental scan targeted any documentation pertaining to the development, implementation, or expansion of eConsult services. We defined eConsult services as asynchronous, directed communication between providers over a secure electronic medium that involved sharing of patient-specific information and sought clarification or guidance regarding clinical care. Although services based in any country were eligible for inclusion, only literature published in English and French were reviewed.

### Literature Review

We conducted a literature search of MEDLINE and EMBASE databases on April 5, 2017 to identify existing eConsult services. Our search strategy built on the keyword combinations and variants used in our previous scan, with modifications to expand the scope beyond Canadian services to include services implemented internationally and focus exclusively on eConsult services (Multimedia Appendix 1).

### Gray Literature Search

Following the literature review, we performed a gray literature search on April 7, 2017 using the Google search engine (Multimedia Appendix 2). If the search yielded >100 hits, the reviewer read through all results until 10 pages (1000 hits) had passed without yielding any information about a new service or the end of the search was reached.

### Key Informant Interviews

Upon completing the literature review and gray literature search, we generated a list of potential interview candidates from among the stakeholders identified in the acquired documents. Potential participants included researchers, health care providers (eg, physicians), and decision makers involved in the development or implementation of an eConsult service. To ensure sufficient breadth of participant experience, we used the maximum variation sampling [10], with relevant factors including the service’s country of origin, technology platform, and host organization. Of note, we did not attempt to contact Canadian services for interviews, as our team had already developed partnerships with all services identified by the scan.

Potential participants were contacted by emails, which were written in English. For services based in countries with majority languages other than English, we generated brief descriptions of the project in their language using Google Translate. A member of our research team (JJ) conducted semistructured interviews by telephone between August 30, 2017 and November 14, 2017 using an interview guide structured around the RE-AIM framework, which assesses a project’s ability to translate research into action using the 5 following categories: reach, effectiveness, adoption, implementation, and maintenance (Multimedia Appendix 3) [11]. The interviewer was a research coordinator with a master’s degree and experience conducting previous qualitative studies; he had no prior relationship with any interview subjects. Interviews began with a brief discussion of the research project’s objectives. All interviews were conducted in English and lasted 20-45 minutes. Interviews were audiorecorded and transcribed verbatim. Participants received a copy of the interview transcript to review and correct if necessary [12].

### Data Analysis

Transcripts were uploaded into NVivo version 11 (QSR International). Team members followed the thematic synthesis approach outlined by Thomas and Harden [13]. One member of the research team (JJ) reviewed the transcripts and developed an initial framework of descriptive and analytical themes. The remaining 6 team members independently reviewed the transcripts using the framework, meeting to discuss progress, identify any disconfirming data, and confirm whether data saturation had been reached. Emerging themes were agreed upon by consensus and amended as needed based on new data.

### Ethics Approval

The Ottawa Health Science Network Research Ethics Board (20120894-01H) and the Bruyère Continuing Care Research Ethics Board (M16-12-052) provided ethics approval for this study.

## Results

### Service Details

**Figure 1:**
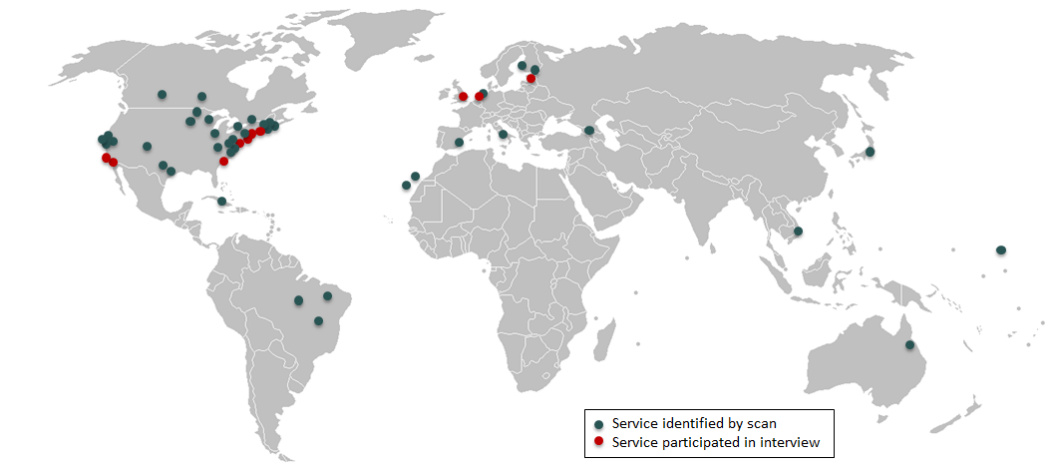
Map of services that were identified by the environmental scan (n=53) and participated in interviews (n=10).

A search of the MEDLINE database returned 262 cases, of which 115 were deemed sufficiently relevant to be reviewed by abstract. A search of the EMBASE database returned 441 cases, of which 172 were sufficiently relevant for abstract review. The results of both searches were combined, resulting in 206 citations after duplicates were removed. A review of these citations revealed 28 distinct eConsult services that met our definition of eConsult (ie, asynchronous platforms that allow PCPs and specialists to discuss a patient’s care). Additional 25 services emerged from the gray literature search, resulting in 53 eConsult services from 17 regions (16 countries plus one international service). The United States had the highest number of identified services (n=28), followed by Canada (n=4), Brazil (n=3), and Spain (n=3). Figure 1 presents a map of all services.

We sent emails to representatives from 49 services (Canadian services, including our own, were excluded from interview recruitment to avoid bias). Representatives from 11 services responded to our emails and completed telephonic interviews. In 2 cases, we held joint interviews with 2 representatives from the service. In another case, 2 separate interviews were conducted about the same service because the initial respondent recommended that we interview another representative. One of the services we interviewed was excluded from our analysis because it was still in its preliminary stages and had not yet developed an eConsult platform. Our final dataset, thus, consisted of 11 interviews with 13 representatives from 10 eConsult services in 4 countries. Respondents held a number of roles, including researchers (n=3), PCPs (n=2), specialists (n=2), managers or directors (n=2), and chief executive or medical or information officers (n=4) and represented a range of service types, varying in size, technology leveraged, and funding model. Table 1 describes the service characteristics.

**Figure 2:**
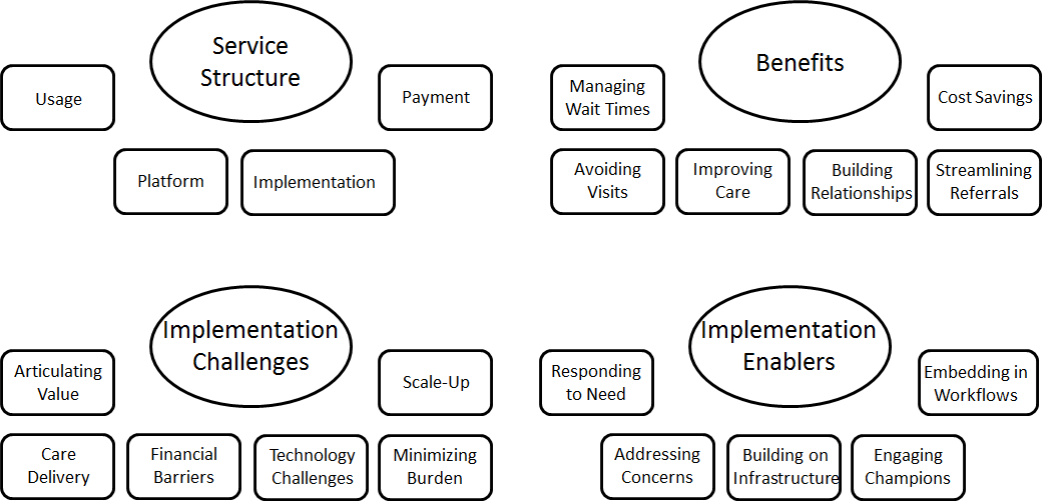
Map of themes and subthemes.

The thematic analysis of the interviews revealed 4 themes as follows: service structure, benefits, implementation challenges, and implementation enablers (Figure 2).

### Service Structure

Respondents discussed a number of issues pertaining to the structure of their eConsult service, including its usage, platform, implementation, and payment.

#### Usage

Usage patterns varied considerably between services, which operated in a range of environments and at vastly different scales. For instance, the Bradford Teaching Hospitals eConsult service offers different single-specialty services, among the largest of which—renal medicine—handles roughly 30 cases a month answered by a single nephrologist, whereas the Veteran’s Health Administration’s New England region processed 90,600 cases in 2015 alone.

**Table 1:** The characteristics of services discussed in telephonic interviews.

Name	Country	Active since	Host organization	Tech platform	Payment model
Estonian Health Information System	Estonia	2011	Government	EMR^a^	Nonprofit
ZorgDomein	Netherlands	2001	Business	EMR	Profit
Bradford Teaching Hospitals	UK	2005	Hospital or clinic	EMR	Nonprofit
AristaMD	US	2014	Business	EMR	Profit
Los Angeles Dept Health Services	US	2012	Government	Web^b^	Nonprofit
NYC Health + Hospitals	US	2015	Hospital or clinic	EMR	Nonprofit
CHC Association of Connecticut	US	2017	Nonprofit	EMR	Nonprofit
Veteran’s Health Administration	US	2011	Government	EMR	Nonprofit
Duke Institute for Health Innovation	US	2016	Research institute	EMR	Nonprofit
RubiconMD	US	2013	Business	Web	Profit

^a^EMR: electronic medical record.

^b^Web: browser-based Web application.

#### Platform

All respondents’ services utilized 1 of 2 main platforms—those integrated into electronic medical records used by participating clinics, and those hosted on the Web and accessed through a Web browser. However, platforms varied considerably within these categories. In some cases, eConsult functioned as part of the referral process, with all referrals automatically made eligible for eConsult. For instance, in the Los Angeles Department of Health Services, “eConsult is the mandated way to request nonurgent, nonemergent outpatient specialty care services from us. There is no other pathway” (Respondent 11). Others, such as RubiconMD, offer “a Web-based and also mobile app-based eConsult platform” (Respondent 9) through which PCPs can submit eConsults if they so choose.

#### Implementation

Respondents’ services were at various stages of implementation, with some well-established services having operated for years, whereas others were only recently launched and still in their pilot phases. Many respondents described the implementation as a gradual process that leveraged grassroots connections, beginning in one instance as “a bottom-up initiative between one family doctor and one hospital” (Respondent 1). Another respondent described the initial service he worked on as operating largely independently alongside a handful of sympathetic providers:

We deliberately went under the radar to start with because we thought there’d be a lot of red tape trying to get this approved. We just thought it was such an obvious thing to bring advantage to patients that we should generate some under-the-radar momentum and enthusiasm and run with that.

Respondent 2

#### Payment

Participating services included for-profit businesses, as well as nonprofit organizations affiliated with universities, hospitals, and regional or national governments. As such, payment mechanisms varied widely based on the objectives of the organization and the health system of the country in which it operated. In US-based systems, the payee was typically a patient’s insurer or, for some populations (eg, safety-net services for low-income individuals, the Veteran’s Health Administration) the state-funded Medicare or Medicaid. In countries with universal health care (eg, the United Kingdom and Estonia), payment came directly from the government. Some services remunerated PCPs and specialists for participating, whereas others—particularly those that had integrated eConsult into the referral process—considered it an extension of the provider’s regular duties and provided no additional or alternate means of payment. For instance, in the Los Angeles Department of Health Services’ system:

[PCPs] don’t see any change in their revenue as a result of using the system or not. The incentive for them to use the system is that this is how they get referrals to their patients.[...]The same goes for the specialists, because in the safety-net systems every doctor just has a flat salary and the whole system is just a flat capitated system.

Respondent 6

### Benefits

Participants described a number of benefits that eConsult provided—managing wait times, avoiding unnecessary visits, improving the quality of care, streamlining the referral process, building provider relationships, and cost savings.

#### Managing Wait Times

Many respondents cited rapid turnaround times as a major benefit of eConsult, noting that their service has helped manage wait times for patients seeking specialist advice, “by doing an eConsult you’re getting all the patients immediate specialist impact by getting someone to weigh in on their care plan” (Respondent 4). Several respondents noted that eConsult provided much-needed relief in areas where wait times were substantial, “There was a pretty significant backlog of referrals that hadn’t been managed at one of the health centers. And so they’re using this pilot as an opportunity to clear out that backlog” (Respondent 7). Respondents stressed how patients benefit from better management of wait times, “It’s also good for the patients as well to get that feedback quickly” (Respondent 2).

#### Avoiding Unnecessary Visits

Several respondents stated that their eConsult service “in many cases helps to avoid a referral” (Respondent 9). Respondents noted the benefit this has for patients, as many of them are able to receive care without the long waits and inconvenience associated with a specialist referral.

#### Improving Quality of Care

Respondents also discussed how eConsult services improve the quality of care patients receive; this improvement was multifaceted and extended beyond the speed of replies and capacity to avoid unnecessary specialist visits. As one respondent noted:

You can improve the quality of care, you can improve the speed of care, you can reduce the cost of care. There are so many aspects associated to teleconsultation.

Respondent 3

While promptness and efficiency emerged as key benefits, respondents argued that eConsult still had value in cases where a face-to-face consultation was required, as it allowed PCPs to better support patients prior to the specialist consultation. As one respondent described:

A third [of cases are] new work, a third avoid a live visit and a third don’t avoid a live visit, but it may actually prepare patients and providers for the live visit better by having trialed a change in medicine before they see the specialist. Or allow the [PCP] to order certain tests that then would be available to the sub-specialist at the time of the visit.

Respondent 8

#### Streamlining the Referral Process

Another benefit of eConsult was its ability to “streamline the referral process” (Respondent 6). One respondent described her service as providing a kind of triage, allowing patients who can be treated at the primary care level to avoid unnecessary visits while freeing up space for those who require face-to-face specialist referrals:

For patients who have higher acuity issues that do need a face-to-face visit, you’re able to identify those patients and expedite them. And because you’re clearing out these lower acuity patients from the waitlist to see the specialist, you’re seeing a huge opening of access to getting face-to-face [appointments]...by giving them earlier face-to-face care by the specialist, you’re not seeing patients sitting for months and months on a waitlist, getting worse, and then having some acute event and ending up in the E.R.

Respondent 4

Another respondent noted that eConsult’s inherent tracking of consultation requests improved accountability by “making sure that every referral gets a specialist’s eyes on it and gets some follow-up” (Respondent 5).

#### Building Provider Relationships and Empowering Primary Care Providers

Several respondents mentioned that the interprovider connections fostered by eConsult can help build relationships between PCPs and specialists. In addition, eConsult can help empower PCPs by providing them with the necessary guidance to perform a broader scope of patient care. As one respondent noted, PCPs who use eConsult “feel that they can provide more [health care services] than expected of them initially” (Respondent 1).

#### Cost Savings

Finally, several respondents discussed eConsult’s ability to save money for patients and the health care system. Respondents noted that a case answered by eConsult costs substantially less than a face-to-face specialist visit:

Keeping the patient at the primary care, that’s the least expensive setting to treat a patient in. [Payers] recognize immediate return on their investment just from avoiding the more expensive specialist visits. And the things that come along with the specialists visits that are often these extremely extensive workups that may or may not be necessary, right. [...] So you’re seeing a reduction in things like E.R. visits and hospital admissions, that’s where gigantic, really, savings come into play.

Respondent 4

### Implementation Challenges

Respondents mentioned several challenges associated with implementing eConsult—articulating service value, ensuring care is effectively delivered, financial barriers, technological challenges, minimizing provider burden, and scale-up.

#### Articulating Service Value

When discussing implementation challenges, nearly all respondents mentioned that they found it difficult to convince stakeholders of eConsult’s value. Often this challenge occurred at the management level, with respondents struggling to secure investment in the implementation from leaders who were skeptical of the service’s efficacy, “the initial challenge was actually convincing people that providers would use this, if it was made available” (Respondent 9). Convincing providers to engage was also sometimes a challenge, though in their case, it was more a question of fighting inertia and getting practitioners to adjust to new methods of delivering care:

The greatest challenge was getting people to think about their work differently. Specialists with the viewpoint that “how can I possibly care for somebody that I haven’t seen face-to-face personally and laid my own hands on them?” Getting them to think about delivering specialty care through this interaction with a primary care physician. Getting PCPs to think about this not as extra work, [but] as an actual patient-centric intervention, because you are setting up a communication with the specialist.

Respondent 11

#### Ensuring Care is Effectively Delivered

According to a few respondents, one of the main challenges with eConsult is ensuring that the service consistently delivers appropriate care. These services tended to be nonprofit organizations that dealt with vulnerable patients and faced limitations in staffing, which at times made it difficult to reach patients and follow up with the advice received through eConsult:

Since we’re a safety-net system there are often concerns with having accurate contact information for patients. Some may change phone numbers, some may not have been comfortable giving us a phone number. [...]Capacity is really an issue for us.

Respondent 5

A respondent from another service noted the particular challenges associated with using eConsult for urgent cases:

If you need urgent specialty care you’re still kind of stuck sitting sometimes in emergency room or begging the specialist, the office, to squeeze somebody in. And it’s hard to get that kind of urgent access. [Respondent 6)

#### Financial Barriers

A few respondents cited financial issues as a challenge to eConsult implementation; these included the logistics of paying providers, as well as securing sufficient funds to implement and run the service. Respondents spoke of the need for buy-in from decision makers capable of financing the service “through a pilot or for some seed money to get it off the ground” (Respondent 9), some of whom were reluctant to support new or unproven programs:

I think the biggest challenge for us has been the politics of some of this with the CEOs who look at this and say ‘yeah, that’s great. But how am I going to get paid? And how am I going to make money from this? Or how am I going to cover my costs?’”

Respondent 7

In addition, one respondent noted that their eConsult service lacked “formal reimbursement mechanisms,” and that it was a challenge to develop “a payment mechanism to support the delivering of eConsult” (Respondent 10). This challenge extended to articulating the value eConsult delivered to patients without an existing business case model.

#### Technological Challenges

Several respondents described technical challenges in eConsult implementation. However, these issues were characterized not as serious issues but as inconveniences or growing pains associated with implementing any new system:

You’re going to run into some things where the information isn’t processing right or there’s something screwy in the EHR or whatever. [...]It’s just a matter of working through those issues.

Respondent 7

This ran counter to some expectations in implementing a technical innovation. One respondent noted that his team “anticipated incorrectly that the main challenge would be technical” (Respondent 10).

#### Minimizing Provider Burden

When discussing their eConsult services, several respondents emphasized the need to minimize the burden of usage it placed on PCPs and specialists. While respondents viewed eConsult as time-saving for the system overall, they noted that adopting the service meant fitting new tasks into extremely busy workflows, an action which some providers resisted:

Whenever you change something there’s always new challenges. [...]PCPs have to make a larger investment in the conversation with the specialists to get their patient in for specialty care, [while specialists] need to have a more robust conversation with the PCPs in order to manage the patient. And so probably our biggest area of complaint or pushback has been the PCP is feeling like it’s more work.

Respondent 11

#### Scale-Up

A few respondents articulated ongoing challenges with scale-up, as their initial services attempt to serve a broader scope of patients over a wider area. Respondents noted that at a larger scale, issues such as payment and service delivery must be more formalized, as structures that worked for a few hundred providers may no longer work with a user base in the thousands.

### Implementation Enablers

Respondents described a number of factors that contributed to the success of their services—responding to an existing need, addressing providers’ concerns and frustrations, building on existing infrastructure, engaging clinical champions, and embedding into provider workflows.

#### Responding to an Existing Need

The most commonly cited enabler for the successful implementation was answering a need that had been articulated by the target population; this need might stem from a policy initiative enacted by regional or national decision makers or from providers frustrated with the current state of affairs. As one respondent described:

We had very long wait times. Many of our specialties had specialty care wait times over 6 months. Some more than a year. There was...the black hole phenomenon where a request would come into us and it would disappear.

Respondent 11

A successful service will…

...build in the right cultural and financial system to make sure that incentives are aligned. So that PCPs have a reason to use it, specialists have a reason to be courteous and timely.

Respondent 6

#### Building on Existing Infrastructure

When designing an eConsult service, many respondents found it advantageous to leverage existing platforms. In many cases, this consisted of an electronic medical records, which had the benefit of already offering a secure digital link between providers and clinics. By harnessing the established infrastructure, respondents were able to build their services at a fraction of the time and cost it would have taken to develop a wholly independent system. One respondent, describing the creation of an eConsult service inside an established network, stated, “I was almost stunned at how straightforward it was” (Respondent 10).

#### Engaging Clinical Champions

Several respondents spoke to the importance of engaging clinical champions early in the implementation process. These individuals were PCPs or specialists who believed strongly in the service, used it often, and advocated on its behalf to their colleagues. As the primary end users of eConsult, health care providers are uniquely positioned to offer feedback on how the service works, and respondents stated that their advocacy lent momentum and legitimacy to the project. In the words of one respondent:

Having those clinical champions as true believers upfront has made all the difference in the world.

Respondent 7

#### Embedding Into Provider Workflows

Several respondents underscored the importance of developing a service that fits “[as] seamlessly as possible into the clinician’s workflow. Because these guys are really strapped for time.” Ease of use was critical to successful adoption, and respondents described taking pains to cut out any extraneous or cumbersome elements from the application:

Understanding the limitations that your teams have on a day-to-day basis and the bottlenecks that they experience has been really critical for us. [...]We had the time to really implement, see how things were going, find out that “x” component here was a few more clicks than it really needed to be, and that was a barrier for staff. And we could resolve that and improve that workflow.

Respondent 5

#### Addressing Providers’ Concerns and Frustrations

To support buy-in from providers, several respondents made a point to seek user feedback regularly throughout the implementation process and address their concerns. Respondents stressed that to get physicians to consider using eConsult, it has to be, at least, as effective and easy to use as the traditional referral-consultation process:

The main selling point for the service has been the commonsense nature of it and the fact that it works well for [PCPs] and it works well for [specialists].

Respondent 2

## Discussion

### Principal Findings

This study found that eConsult services are being implemented in countries around the world. Services can take a number of different forms, with variations in scope, technology platform, financial structure, and engagement strategy. They did not come predominantly from any one sector, emerging as private companies, research pilots, government initiatives, and extensions of existing hospitals or health care clinics. Despite these differences, respondents frequently described facing similar barriers in their implementation and cited common factors that enabled the successful implementation and growth of their services. Gaining interest from stakeholders, ensuring the service effectively meets its stated aims, and securing financial support were among the most frequently cited barriers, while engaging clinical champions, building on existing infrastructure, and addressing an existing need emerged as the main enablers of success.

### Limitations

This study has several limitations. Of 53 services identified by the environmental scan, only 11 participated in interviews (10 of which were included). Services from the United States are disproportionately represented, making generalization to other countries more difficult; this limitation is exacerbated by our ability to conduct interviews in only 2 languages (English and French). Although the effort was made to contact all services regardless of their location, our lack of fluency in other languages likely hindered our ability to recruit participants. In addition, all health care providers who participated in this study were physicians. As such, the views of other eConsult users (eg, nurse practitioners) may not have been reflected.

### Comparison With Prior Work

Among enablers, addressing an existing need was often described as a particularly important step. All services in this study emerged to address a common problem of poor access to specialist care, with individual approaches tailored to address each service’s target population. This approach reflects our own experience with the Champlain BASE eConsult service. Our team created eConsult as a direct response to excessive wait times for specialist care, which remains a significant and ongoing problem in Canada. A 2016 survey by the Commonwealth Fund assessed 11 countries on measures of the health care quality, including access to care. Canada ranked last on wait times for specialist care, with 56% of patients waiting ≥4 weeks for an appointment versus an average of 36% [14]. The severity of this issue drove the Champlain BASE eConsult service’s implementation in our region. Likewise, a number of respondents in this study built their own services around the needs of their communities. For instance, in the Commonwealth Fund survey cited above, the United States fared relatively well on the metric of specialist wait times—ranking third out of 11 participants—but faced a number of substantial barriers related to equity and cost of care [14]. As such, several of the United States-based services in this study developed their programs with a lens toward improving equity. Notably, several were “safety net services” specifically designed to help vulnerable individuals who lacked private insurance.

Encouragingly, eConsult is a flexible and multifaceted solution and has shown itself to be well-positioned to address the wide range of access issues presented by communities in different countries. Respondents witnessed a wide range of benefits of their eConsult services, including their ability to avoid unnecessary specialist visits, improve the overall quality of care, reduce costs, and improve communication between providers. These assertions are supported by the literature, which has reported many of the same benefits for eConsult services [7,8]. A systematic review conducted in 2015 identified 27 peer-reviewed papers discussing eConsult services and found high levels of provider satisfaction (70%-95%), quick response times (<3 days in most cases), and avoidance of unnecessary referrals [7]. A systematic review by our team found similar results, as well as some evidence of reduced costs [8].

### Future of eConsult

The breadth of eConsult services now operating worldwide suggests a promising future for this model of health service delivery. In many cases, regional health authorities have integrated eConsult into the fabric of the health system, making it a mandatory component of the referral-consultation process. Other systems, including Champlain BASE, are supplemental and voluntary, relying on the provider and patient interest to drive engagement. While barriers to the eConsult’s expansion exist and must be addressed [15], the overall picture is encouraging, as evidenced by the experiences highlighted in this study. Furthermore, our efforts at the expansion have been highly successful; the service is currently expanding province-wide, and the College of Family Physicians of Canada recently released a statement identifying eConsult as a standard of practice.

The growing focus on eConsult as a method of improving patients’ access to care can be seen as an extension of the Patient’s Medical Home, a model of health service delivery that emphasizes that each patient should have a dedicated family practice that serves as “the central hub for the timely provision and coordination of a comprehensive menu of health and medical services patients need” [16].

The goal of the Patient’s Medical Home fits naturally into eConsult, as such services allow PCPs to take a more central role in their patients’ care. By using eConsult, PCPs are often able to gain the guidance they need to treat patients themselves when they would otherwise have referred them, and its capacity for direct interprovider communication improves care coordination and reduces the risk of cases being forgotten or recommendations lost.

### Conclusions

eConsult services have emerged in a variety of countries and health system contexts worldwide. Structure, platform, and delivery model varied, but the services consistently demonstrated improved access and high levels of satisfaction. Respondents encountered several barriers to implementation but were able to overcome them by addressing an existing need and working with engaged clinician leaders. Lessons learned from this group will be helpful for those looking to implement an eConsult service in their own jurisdictions.

## Appendix

Multimedia Appendix 1 Search strategy matrix for literature review.

Multimedia Appendix 2 Strategy used for gray literature search.

Multimedia Appendix 3 Semistructured interview guide.

## Abbreviations

BASE: Building Access to Specialists through eConsultation
eConsult: electronic consultation
eReferral: electronic referral
PCP: primary care provider
