# Supplementary material for: Electronic Consultation Services Worldwide: Environmental Scan
Source: J Med Internet Res. 2018 Dec 21;20(12):e11112. doi: 10.2196/11112 (PMC6320413; doi:10.2196/11112)
Supplement: Multimedia Appendix 1 [file jmir_v20i12e11112_app1.pdf]

Appendix A. Search Strategy matrix for literature review

	**Consult**	**Consultation**
**Electronic**	x	x
**E-**	x	x
**e**	x	x
**Web Based**	x	x
**Web-based**	x	x
**Internet**	x	x
**Internet-Based**	x	x
**Internet based**	x	x
**Online**	x	x
**Virtual**	x	x
**Computer-based**	x	x
**Computer based**	x	x
**Intranet**	x	x
**Store-and-forward**	x	x
