# Supplementary material for: Electronic Consultation Services Worldwide: Environmental Scan
Source: J Med Internet Res. 2018 Dec 21;20(12):e11112. doi: 10.2196/11112 (PMC6320413; doi:10.2196/11112)
Supplement: Multimedia Appendix 2 [file jmir_v20i12e11112_app2.pdf]

Appendix B. Strategy used for grey literature search

electronic consultation	AND	(healthcare or health care)
Systematically substituted with the following terms:<br>• e-consultation<br>• eConsultation<br>• eConsult		
