# Supplementary material for: Electronic Consultation Services Worldwide: Environmental Scan
Source: J Med Internet Res. 2018 Dec 21;20(12):e11112. doi: 10.2196/11112 (PMC6320413; doi:10.2196/11112)
Supplement: Multimedia Appendix 3 [file jmir_v20i12e11112_app3.pdf]

Appendix C. Semi-structured interview guide

Program Description	1. Can you describe the electronic system?<br>2. Can you describe your involvement with the system?<br>3. What funding structure existed to support the system upon implementation, and how has that funding structure changed since that time?<br>4. Is the system still currently operating?<br>a) If not, how long was it operational and why is it no longer?<br>b) If so, how long has it been operational?
Reach	5. How widely used is/was the system (number of practices, health regions, percent of total practices in province)?
Effectiveness	6. How has the electronic system impacted the overall referral process?<br>7. What aspects of the system, if any, have the users found most useful?<br>8. What aspects of the system, if any, have users found less useful and/or complained about?<br>9. What elements of the system have you changed since its original implementation, and why did you make those changes?
Adoption	10. Were physician-users resistant to adopting the system?<br>a) If so, can you expand upon the reasons for this?<br>11. Can you suggest any strategies that would have increased the uptake?<br>12. Has the adoption improved since the initial implementation phase? If so, can you provide some reasons why?
Implementation	13. What was the strategy for implementing the system?<br>14. How much training was required before users could interact with the system?<br>15. Can you describe any of the key aspects that facilitated implementation of the system?<br>16. Can you describe the relevant barriers to implementation of the system?
Maintenance/ Sustainability	17. Can you suggest any strategies to ensuring the sustainability of an e-consult/e-referral system?
Thank you for taking part in this interview	
